# Effect of elevated CO_2_ and spectral quality on whole plant gas exchange patterns in tomatoes

**DOI:** 10.1371/journal.pone.0205861

**Published:** 2018-10-18

**Authors:** Jason Lanoue, Evangelos D. Leonardos, Shalin Khosla, Xiuming Hao, Bernard Grodzinski

**Affiliations:** 1 Department of Plant Agriculture, University of Guelph, Guelph, Ontario, Canada; 2 Harrow Research and Development Centre, Agriculture & Agri-Food Canada, Harrow, Ontario, Canada; 3 Ontario Ministry of Agriculture, Food and Rural Affairs, Harrow, Ontario, Canada; Universidade Federal de Vicosa, BRAZIL

## Abstract

In controlled environment plant production facilities, elevating either light or CO_2_ levels generally has led to increased biomass and yield due to enhanced canopy photosynthesis. Today, advancements in light-emitting diodes (LEDs) have made this technology a viable option for both supplementary lighting in greenhouses and a sole lighting source in controlled environment chambers. Our study used tomato plants grown under both ambient CO_2_ (AC) and elevated CO_2_ (EC) conditions then exposed them to various CO_2_ and lighting treatments during both whole plant and leaf level measurements. Plants grown under EC reached the first flower developmental stage 8 days sooner and were approximately 15cm taller than those grown under AC. However, under AC plants had more leaf area while their dry weights were similar. Of note, under EC chlorophyll *a* and *b* were lower, as were carotenoids per unit leaf area. Whole plant analyses, under all CO_2_ challenges, showed that plants exposed to high-pressure sodium (HPS), red-blue LED, and red-white LED had similar photosynthesis, respiration, and daily carbon gain. Under different light qualities, day-time transpiration rates were similar among CO_2_ conditions. Day-time water-use efficiency (WUE) was higher in plants grown and exposed to EC. Similarly, WUE of plants grown under AC but exposed to short-term elevated CO_2_ conditions was higher than those grown and tested under AC during all light treatments. Under all CO_2_ conditions, plants exposed to red-white and red-blue LEDs had lower WUE than those exposed to HPS lighting. Assessing alterations due to CO_2_ and light quality on a whole plant basis, not merely on an individual leaf basis, furthers our understanding of the interactions between these two parameters during controlled environment production. Principle component analyses of both whole plant and leaf data indicates that increasing CO_2_ supply has a more dramatic effect on photosynthesis and WUE than on transpiration.

## Introduction

An increase in atmospheric CO_2_ is now inevitable, with some predictions indicating concentrations in excess of 1100ppm by the end of the century [1]. While plants in natural environments are just beginning to be exposed to increasing CO_2_ concentrations, elevated CO_2_ (EC) has been a staple fertilization method in controlled environment production with varied results [2–3]. Within high value greenhouse crops, such as tomatoes, growth under EC has led to increases in both biomass production and yield [4–6].

Light plays a critical role in production as it too increases biomass accumulation when intensity is increased [7]. Recent advancements in light-emitting diode (LED) technology, including lower production cost and increased energy efficiency, have made them a viable alternative to high pressure sodium (HPS) lighting for both sole and supplemental lighting [8–10]. A specific advantage of LEDs is their ability to generate wavelength specific lighting. Plants of all species have been observed to have different responses in morphology, primary gas exchanges, and gene expression to illumination with varying wavelengths [11–15].

Whole plant gas exchange analysis allows for a non-destructive estimation of daily growth patterns and water loss [15–17]. The use of whole plant gas exchange systems provides additional information regarding leaf age and light interception within a plant canopy which is often not apparent during leaf analysis [15–19]. Accounting for differences in plant and leaf morphology is especially important when trying to compare plants which were grown under different conditions known to alter plant architecture, such as light quality and CO_2_ concentration [13, 20–23]. Furthermore, obtaining whole plant data allows for a greater understanding of the interactions between environmental stimuli. This, in turn, can allow for the translation of information to controlled environment production and climate regulation related to common inputs such as CO_2_ and light [24–25].

The use of whole plant analysis allows for the study of net carbon exchange rate (NCER), transpiration, and water-use efficiency (WUE) on plants at similar developmental stage and size with inherently different morphologies due to their growth conditions. Our study, by design, used plants grown under either ambient CO_2_ (AC) or elevated CO_2_ (EC). Whole plant and leaf CO_2_ and H_2_O gas exchanges were then analyzed at a similar developmental stage under lights of differing spectral quality and various CO_2_ conditions. Our primary objective was to assess how primary gas exchanges of CO_2_ and H_2_O of tomato plants with different canopy architecture are affected by light quality and CO_2_ concentration and how whole plant response compare to leaf responses.

## Materials and methods

### Plant material and growth conditions

Seeds of *Solanum lycopersicum* cv. “Bonny Best” (William Dam Seeds; Dundas, ON, Canada) were sown into 60 cavity potting trays in Sungro professional growing mix #1 (Soba Beach, AB, Canada) and placed in growth chambers (GC-20 Bigfoot series, Biochambers, Winnipeg, MB, Canada). Temperature was set to 22/18°C (d/n) with a 16/8h photoperiod. Plants were illuminated with 300±25 μmol m^-2^ s^-1^ of photosynthetically active radiation (PAR) from compact fluorescent lights (Sylvania Pentron 841 HO Ecologic, Wilmington, MA, USA). Relative humidity was maintained at 60±10%. Growth chambers contained either AC (400±10 μL L^-1^) or EC (1000±20 μL L^-1^). Growth chamber CO_2_ conditions were rotated periodically to eliminate chamber bias. Plants were watered with fertilizer as needed (20-8-20; pH = 6, EC = 2.3mS cm^-1^). For both whole plant and leaf experiments, plants grown under AC were analyzed when they were 40–46 days after sowing and plants grown under EC were analyzed when they were 33–38 days after sowing. This was done in order to use plants which were at the first flower developmental stage. This stage was chosen as it represents the transition point for solely vegetative growth to a combination of vegetative and reproductive growth. Furthermore, this stage allowed for a defined point in which plants grown under different CO_2_ conditions were at the same developmental stage. All experiments were performed in the Controlled Environment Systems Research Facility at the University of Guelph.

### Growth analysis

#### Diurnal patterns of whole plant gas exchanges

The whole plant gas exchange system is identical to that used in Lanoue et al. [15]. Gas exchange measurements were made by sampling each chamber for 90s, cycling through all 6 chambers every 9-minutes throughout day/night periods. Two chambers were illuminated with high pressure sodium (HPS) lights (Agrolite XT; Phillips Lighting, Markham, ON, Canada), two chambers were illuminated with red-blue (RB; LsPro VividGro V1 Grow Fixture; Lighting Science Group Company (LSGC) Warwick, RI, USA) LEDs, and two chambers were illuminated with red-white (RW; LSGC) LEDs (Figure A in S1 File). Light treatments were rotated between chambers to remove chamber bias.

Ambient CO_2_ during analysis was 400±10μL L^-1^ and EC during analysis was 1000±10μL L^-1^. Plants grown under AC conditions were analyzed under either AC or short-term exposure to elevated CO_2_ (SEC). Plants grown under EC conditions were analyzed only under EC. During EC and SEC analysis, night time CO_2_ levels were 400±10μL L^-1^. Plants which were grown under fluorescent lighting were placed in the chambers the day before around 15:00:00 and measurements used for the calculations of gas exchange were taken from the following day/night period. Lights were set to 1000±10 μmol m^-2^ s^-1^ at the top of the plant canopy as determined by a Li-COR quantum sensor with a photoperiod of 16/8h. Temperature and relative humidity were 22/18°C and 60±5% respectively.

#### Biomass partitioning (destructive analysis)

Following each whole plant experiment, plants were removed from the chambers and leaf area was measured using a leaf area meter (Li-COR 3100, Li-COR Inc. Lincoln, NE, USA). The roots were washed free of dirt then plant material (leaves, stems, and roots) were partitioned and dried in an oven for 48h at 80°C then weighed.

#### Chlorophyll content

Prior to entering the whole plant gas exchange system, 6 SPAD measurements were taken from each plant. Two SPAD measurements were taken from each of the upper, middle, and lower ranked leaves. SPAD measurements were similar between leaf ranks and thus, data was pooled. This protocol was repeated at the end of each experimental run. SPAD readings were not altered during whole plant analysis allowing for pooling of reading taken before and after the experiment run (12 measurements in total). SPAD measurements were then converted to chlorophyll content using correction equations generated by spectrophotometer pigment analysis in which chlorophyll concentrations were assigned to SPAD values. Chlorophyll correction curves were generated by extracting leaf punches in 100% dimethyl formamide for 28h at 4°C. Samples were than analyzed at 663.8nm, 646.8nm, and 480nm wavelengths using a spectrophotometer. Concentrations of chlorophyll *a*, *b*, and carotenoids were determined via equations from Porra et al. [26] and Wellburn [27]. Correlation equations were determined for plants grown under both AC and EC.

### Leaf gas exchange measurements

The fifth most fully expanded leaf was placed in the chamber of a Li-COR 6400 (Li-COR Inc. Lincoln, NE, USA) which was fitted with a 2cm x 3cm clear top chamber. The leaf temperature within the chamber was held at 22°C with a relative humidity of 55–65%. CO_2_ conditions included AC, EC, SEC, and short-term ambient CO_2_ (SAC) in which plants were grown under elevated CO_2_ than analyzed at ambient CO_2_ levels. Lights used to generate the leaf gas exchange curves were specially designed LED flood lights (PAR 38, LSGC) as well as an HPS luminary. LEDs produced the following peak wavelengths: red (660nm), blue (440nm), orange (595nm), green (500nm), white, red-blue, or red-white (Figure A in S1 File). Three leaves, each from a different plant, were used for each light treatment. Light curves began at a high light intensity and decreased incrementally which follows the procedure from Evans & Santiago [28]. At each light level, the photosynthetic rate was allowed to reach steady-state then a 2-minute period was averaged to produce values for that light level. Of note, for plants under the CO_2_ condition SAC, a light curve was not performed, but a measurement at 500 μmol m^-2^ s^-1^ light level was obtained.

### Statistical analysis

All statistics were performed using SAS studio 3.5. Means comparisons were done using a one-way ANOVA with a Tukey Kramer adjustment at p<0.05. Principle component analysis (PCA) [29–30] was applied to determine the relationship between CO_2_ and H_2_O gas exchange under different light qualities and CO_2_ concentrations for both whole plant and leaf data. For both whole plant and leaf PCA, the analysis was performed using daily averages from each individual experimental run (Figure B and C in S1 File).

## Results

**Fig 1:**
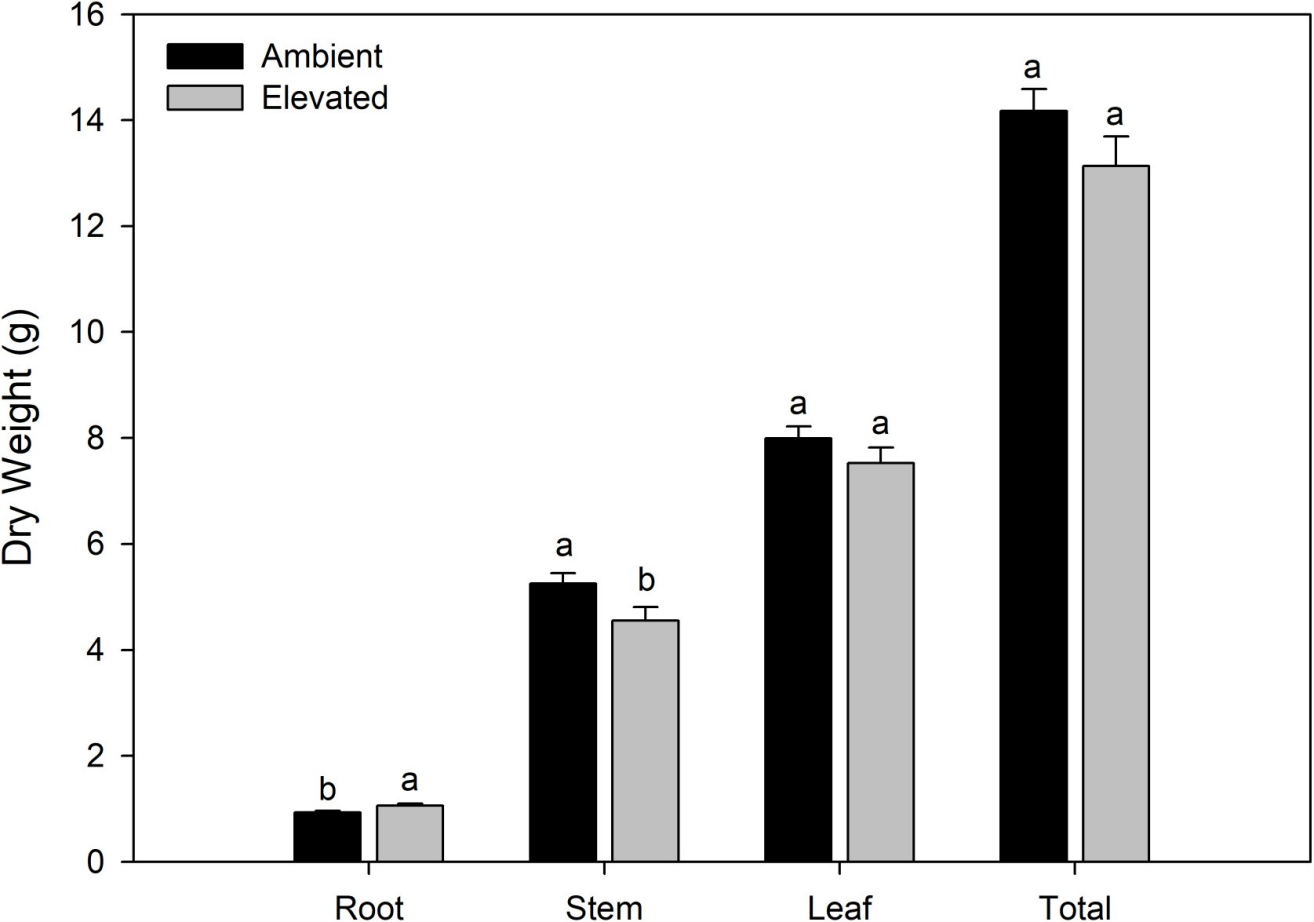
Biomass partitioning of plants grown under AC or EC. Different letter groups (a,b) represent statistical differences within each plant section at p<0.05 with n = 42.

Plants grown under EC reached the flowering stage on average 8 days sooner than did plants grown under AC conditions. Upon destructive biomass harvest, plants grown under EC conditions produced a larger root dry mass than plants grown at AC (Fig 1). Plants grown under AC conditions produced a larger stem dry mass than plants grown under EC (Fig 1). However, under both conditions, plants had similar total dry matter at their respective first flower developmental stage (Fig 1).

Plants which were grown under EC conditions were taller at the time of whole plant analysis (Table 1). An increased leaf area was determined from plants grown under AC conditions (Table 1). However, plants grown under EC had a higher specific leaf mass (Table 1). Furthermore, plants grown under EC also produced a higher root:shoot (Table 1).

**Table 1:** Morphological parameters of plants grown under AC and EC.

Growth Conditions	Plant Height (cm)	Leaf Area (cm^2^)	Specific Leaf Mass (g m^-2^)	Root:Shoot
**Ambient (400 μL L**^**-1**^**)**	26.87(0.55)^b^	1992.06(58.32)^a^	40.74(0.65)^b^	0.073(0.0002)^b^
**Elevated (1000 μL L**^**-1**^**)**	41.41(0.63)^a^	1512.73(44.47)^b^	50.49(0.82)^a^	0.097(0.0004)^a^

^a, b^ statistical differences within each parameter at p<0.05 with n = 20 for plant height and n = 42 for leaf area, specific leaf mass, and root:shoot.

Pigment analysis determined that short-term illumination under different spectral quality did not alter pigment content and thus SPAD readings taken prior to and after each experiment were pooled. Plants grown under EC had lower levels of chlorophyll *a*, chlorophyll *b*, total chlorophyll, chlorophyll *a*:*b*, and carotenoids compare to plants grown under AC conditions (Table 2).

**Table 2:** Pigment analysis of plants grown under AC and EC.

Growth Conditions	Chlorophyll *a* (μg cm^-2^)	Chlorophyll *b* (μg cm^-2^)	Chlorophyll *a*+*b* (μg cm^-2^)	Chlorophyll *a*:*b*	Carotenoids (μg cm^-2^)
**Ambient (400 μL L**^**-1**^**)**	43.06(0.47)^a^	11.91(0.13)^a^	54.97(0.60)^a^	3.62(0.0014)^a^	10.38(0.095)^a^
**Elevated (1000 μL L**^**-1**^**)**	34.03(0.43)^b^	9.55(0.14)^b^	43.58(0.57)^b^	3.58(0.010)^b^	8.50(0.078)^b^

^a, b^ statistical differences within each parameter at p<0.05 with n = 42.

**Fig 2:**
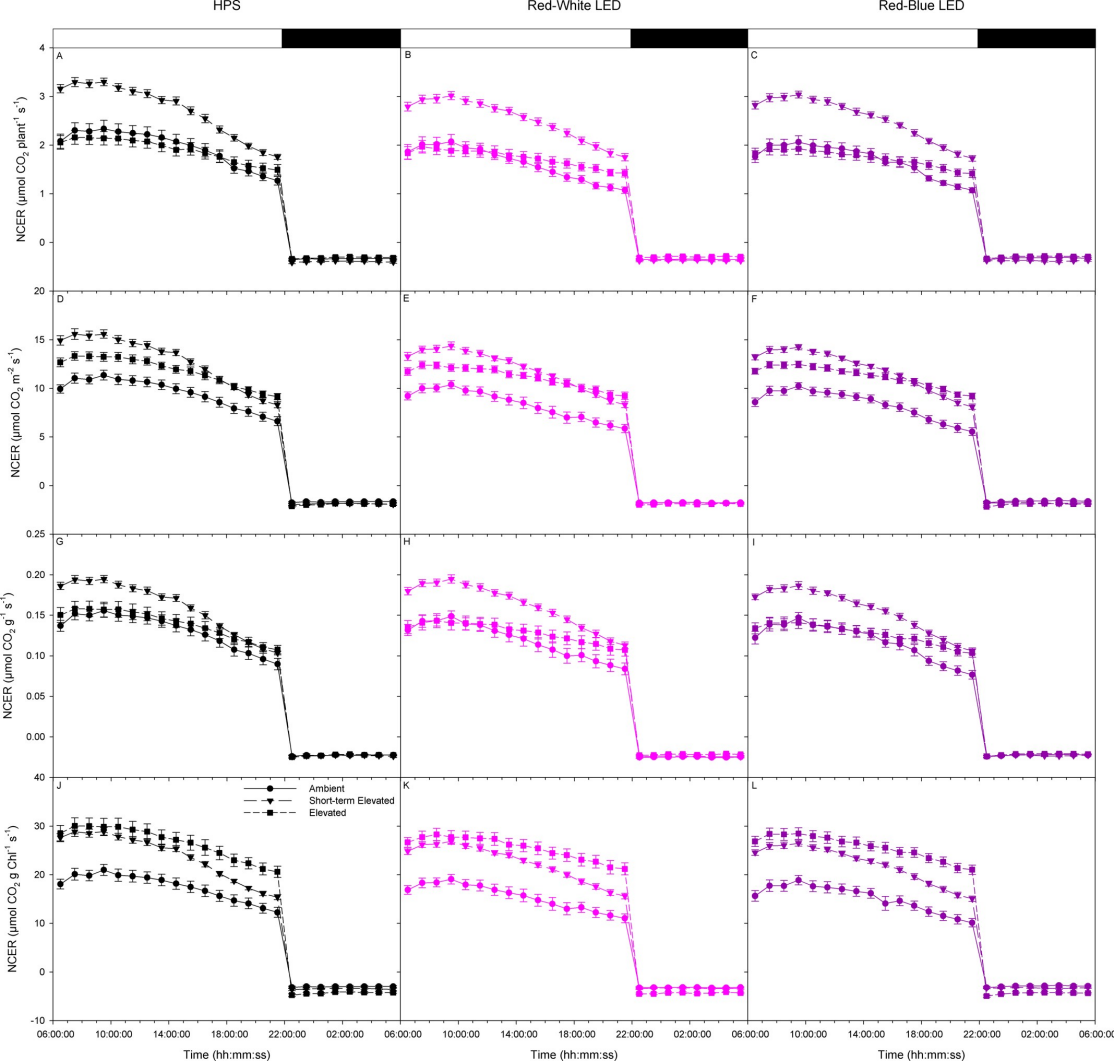
Diurnal patterns of whole plant CO_2_ gas exchange of tomatoes at the first flower developmental stage grown at either ambient or elevated CO_2_. Plant grown under AC or EC conditions were analyzed under the same CO_2_ conditions as well as AC plants analyzed under short-term elevated CO_2_ (SEC) conditions. Plants were analyzed under either HPS (panels A, D, G, & J), red-white LED (panels B, E, H, & K), or red-blue LED (panels C, F, I, & L). Panels A-C are NCER normalized on a plant basis, panels D-F are NCER normalized on a leaf area basis, panels G-I are NCER normalized on a total dry weight basis, and panels J-L are NCER normalized on a chlorophyll basis. Each point and error bars represent the averages and standard error of n = 14 respectively.

Fig 2 displays the primary diurnal whole plant NCER of tomatoes plants which were grown under either AC or EC conditions. Plants were then exposed to either AC, EC, or SEC conditions during whole plant analysis under either HPS (Fig 2A, 2D, 2G and 2J), red-white LED (Fig 2B, 2E, 2H and 2K), or red-blue LED (Fig 2C, 2F, 2I and 2L). Net carbon exchange rates were then expressed on a plant basis (Fig 2A, 2B and 2C), a leaf area basis (Fig 2D, 2E and 2F), a dry weight basis (Fig 2G, 2H and 2I), and a chlorophyll basis (Fig 2J, 2K and 2L). This was done in order to compare plants on the same metric while taking into account any intrinsic differences in morphologies brought about during growth under different CO_2_ conditions.

Under all CO_2_ conditions and light treatments, day-time (06:00:00–22:00:00) NCER followed similar patterns, being steady during the morning hours and then decreasing during the afternoon hours until the end of the photoperiod (Fig 2). Notably, under SEC, the drop-in day-time NCER was observed to be more drastic than under EC conditions (Fig 2). Under all CO_2_ and light conditions, night-time patterns of respiration were similar (Fig 2).

**Fig 3:**
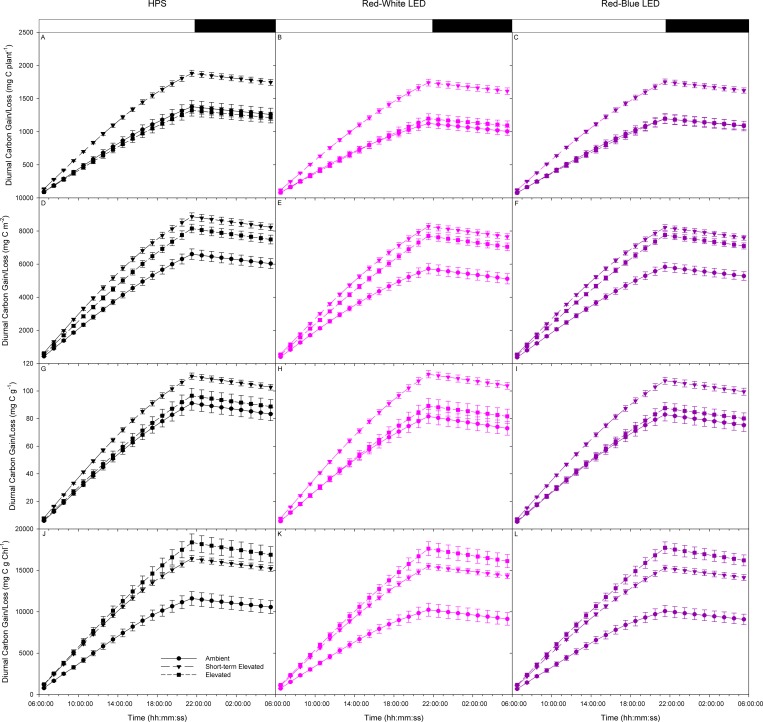
Diurnal patterns of whole plant carbon gain/loss of tomatoes at the first flower developmental stage grown at either ambient or elevated CO_2_. Plant grown under AC or EC conditions were analyzed under the same CO_2_ conditions as well as AC plants analyzed under short-term elevated CO_2_ (SEC) conditions. Plants were analyzed under either HPS (panels A, D, G, & J), red-white LED (panels B, E, H, & K), or red-blue LED (panels C, F, I, & L). Panels A-C are NCER normalized on a plant basis, panels D-F are NCER normalized on a leaf area basis, panels G-I are NCER normalized on a total dry weight basis, and panels J-L are NCER normalized on a chlorophyll basis. Each point and error bars represent the averages and standard error of n = 14 respectively.

The integration of the data presented in Fig 2 is presented in Fig 3. This data represents the carbon gained by the plant during the light period via carbon assimilation and the subsequent carbon lost during the night period due to respiration (Fig 3). On both a plant and dry weight basis, plants under the SEC condition accumulated the most carbon by the end of the photoperiod under all lighting conditions (Fig 3A–3C, 3G–3I). When expressed on a leaf area basis, plants under both EC and SEC conditions showed an increase in carbon accumulation under all light treatments compared to the AC condition (Fig 3D–3F). Although on a plant basis the carbon gained during the photoperiod is similar between EC and AC plants, the decrease in leaf area of EC plants compared to AC plants (Table 1) causes the increase in carbon gained under the EC condition (Fig 3D–3F). On a chlorophyll basis, similar to the NCER data in Fig 2J–2L, plants under both EC and SEC conditions gained more carbon during the photoperiod under all light conditions than plants under AC conditions (Fig 3J–3L). Similar to the decrease in leaf area, acclimation to EC caused a decrease in the amount of chlorophyll (Table 2) and thus, on a chlorophyll basis, an increase in carbon accumulation during the photoperiod was observed under all light treatments from EC plants compared to plants under AC (Fig 3J–3L). Consistent with the results obtained in Fig 2, under all CO_2_ and light treatments, the amount of carbon loss during the night period was similar (Fig 3).

**Fig 4:**
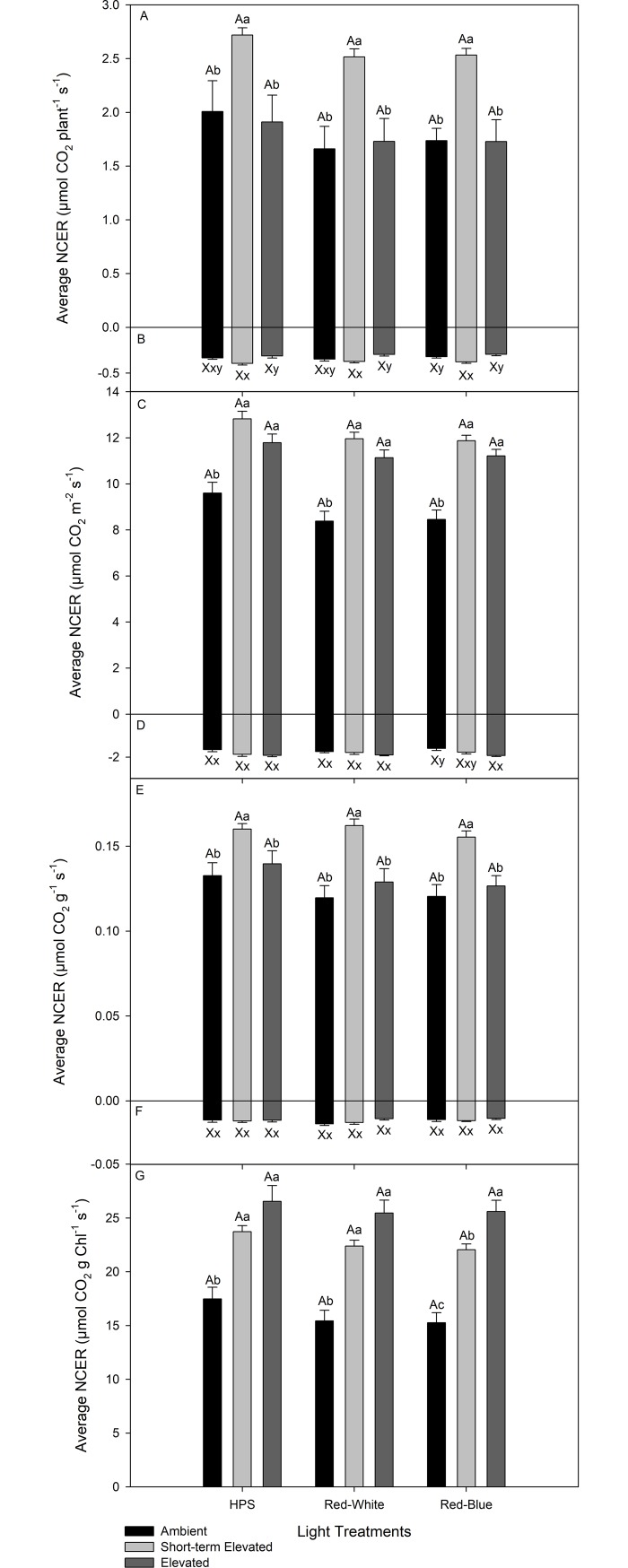
**Day and night time average NCER normalized on a plant (panel A–photosynthesis; B–respiration), leaf area (panel C–photosynthesis; D–respiration), dry weight (panel E–photosynthesis; F–respiration), and chlorophyll basis (panel G–photosynthesis).** Different upper-case letter groups (A, B, C or X, Y) represent statistically different values between light treatments at the same CO_2_ analysis conditions within a panel at p<0.05 where n = 14 for day and night-time averages respectively. Different lower-case letter groups (a, b, c or x, y) represent statistically different values within the same light treatments at different CO_2_ analysis conditions within a panel at p<0.05 where n = 14 for day and night-time averages respectively.

Day-time average whole plant photosynthetic rate was highest when plants were exposed to SEC on a plant basis under all light conditions (Fig 4A). Average day-time photosynthetic rates were similar between lights within each CO_2_ condition on a plant basis (Fig 4A). Under all light conditions, plants exposed to SEC produced higher night-time respiration rates than did plants grown and analyzed under EC (Fig 4B). When plants were analyzed under the RB LED, plants exposed to SEC produced higher respiration rates than plants grown and analyzed under AC (Fig 4B).

On a leaf area basis, day-time average whole plant photosynthetic rates were highest when plants were grown and analyzed under EC and when plants were exposed to SEC under all light conditions (Fig 4C). Within each CO_2_ condition, lights produced similar average day-time photosynthetic rates (Fig 4C). Within each CO_2_ condition, night-time respiration rates were similar between light treatments (Fig 4D). When analyzed under HPS and RW LED, average respiration rates were similar regardless of CO_2_ conditions (Fig 4D). When analyzed under RB LED, plants analyzed under EC produced higher average respiration rates than plants analyzed under AC (Fig 4D).

Plants exposed to SEC produced the highest day-time average photosynthetic rate compared to those grown and analyzed under either AC or EC under all light treatments when normalized or a dry weight basis (Fig 4E). Within each CO_2_ conditions, all light treatments produced similar average day-time photosynthetic rates (Fig 4E). Similar night-time respiration rates were observed under all CO_2_ and light treatments (Fig 4F).

When normalized on a total chlorophyll content basis, both plants grown under EC and analyzed under EC and SEC produced the highest average day-time photosynthetic rates under all light treatments (Fig 4G). When analyzed under RB LED, plants grown and analyzed under EC also produced a higher average photosynthetic rate than did plants exposed to SEC (Fig 4G).

**Fig 5:**
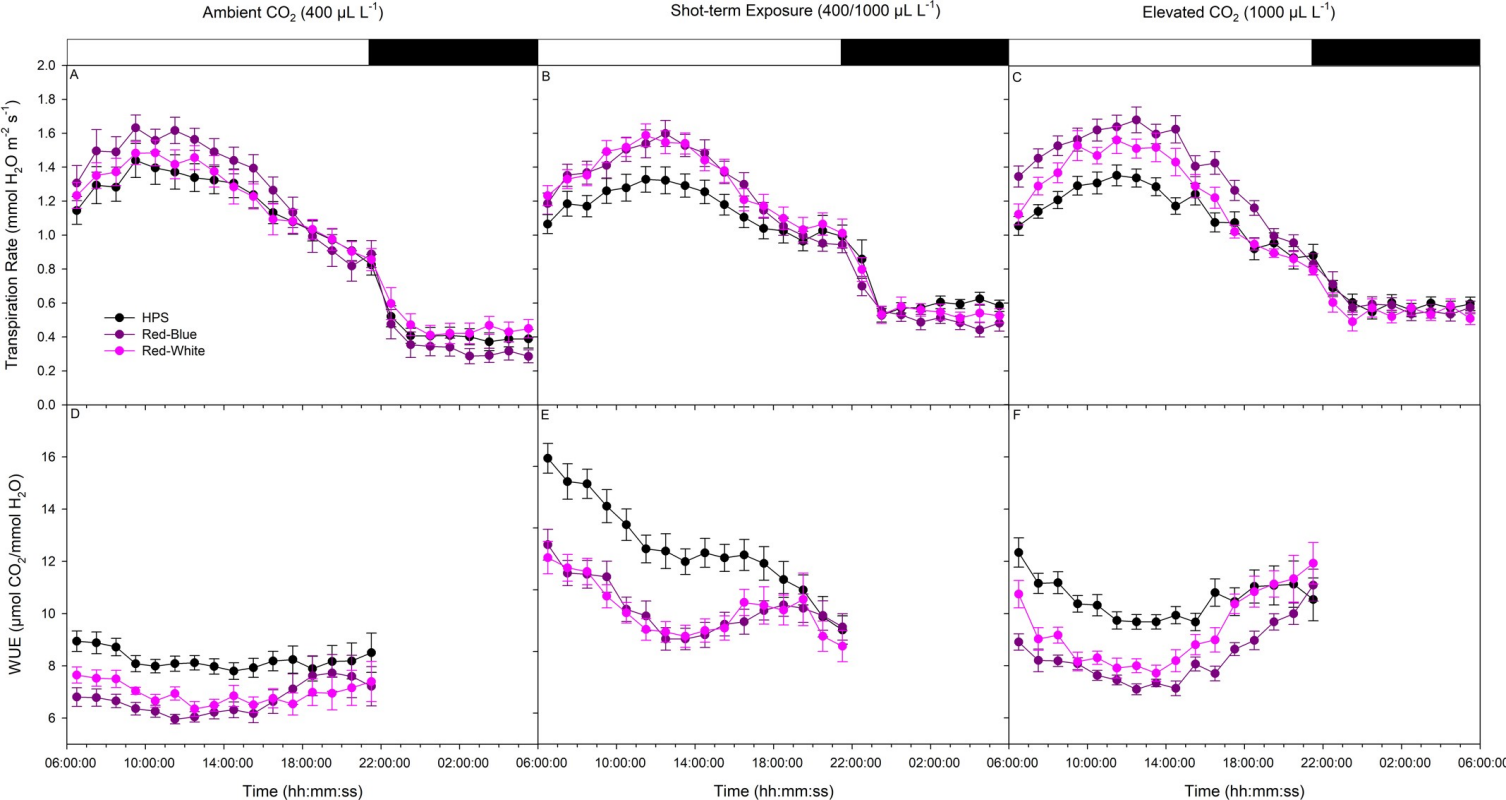
Diurnal patterns of whole plant H_2_O gas exchange of plants grown at either AC or EC. Plants were either exposed to the same CO_2_ concentration as their growth condition (panels A, D–ambient; panels C, F–elevated) or SEC (panels B, E). Panels A-C are whole plant transpiration rates and panels D-F are whole day-time WUE. Each point and error bars represent the averages and standard error of n = 14 respectively.

Diurnal patterns of whole plant transpiration rate followed similar patterns under all CO_2_ and light treatments (Fig 5A, 5B and 5C). Day-time transpiration rates increased from the start of the photoperiod and reached a maximum around midday (12:00:00–14:00:00) than decreased until the end of the photoperiod (22:00:00) under all light and CO_2_ treatments (Fig 5A, 5B and 5C). Night-time transpiration rates were lower than day-time conditions under all treatments and remained steady during the night period (Fig 5A, 5B and 5C).

Day-time patterns of WUE for plants grown under and analyzed under both AC and EC followed similar patterns throughout the photoperiod (Fig 5D and 5F). WUE were highest at the start of the photoperiod, decreased to a minimum around midday (12:00:00–14:00:00) than increased to levels comparable to the start of the day, at the end of the photoperiod (Fig 5D and 5F). Of note, under both CO_2_ conditions, plants illuminated with RB and RW LEDs had lower WUE during the beginning and middle of the day than plants illuminated with HPS lighting (Fig 5D and 5F). However, at the end of the day, all light treatments under AC and EC converged and similar efficiencies were observed (Fig 5D and 5F). Interestingly, patterns of day-time WUE were observed to be different under SEC than the other two CO_2_ conditions (Fig 5E). WUE was still determined to be highest at the start of the day and decrease until midday (Fig 5E). However, instead of increasing back to efficiencies similar to the beginning of the photoperiod, only a slight increase, followed by a decrease until the end of the photoperiod was observed (Fig 5E). Unlike both AC and EC conditions, end of photoperiod WUE in SEC plants were among the lowest efficiencies observed during the photoperiod (Fig 5E).

**Fig 6:**
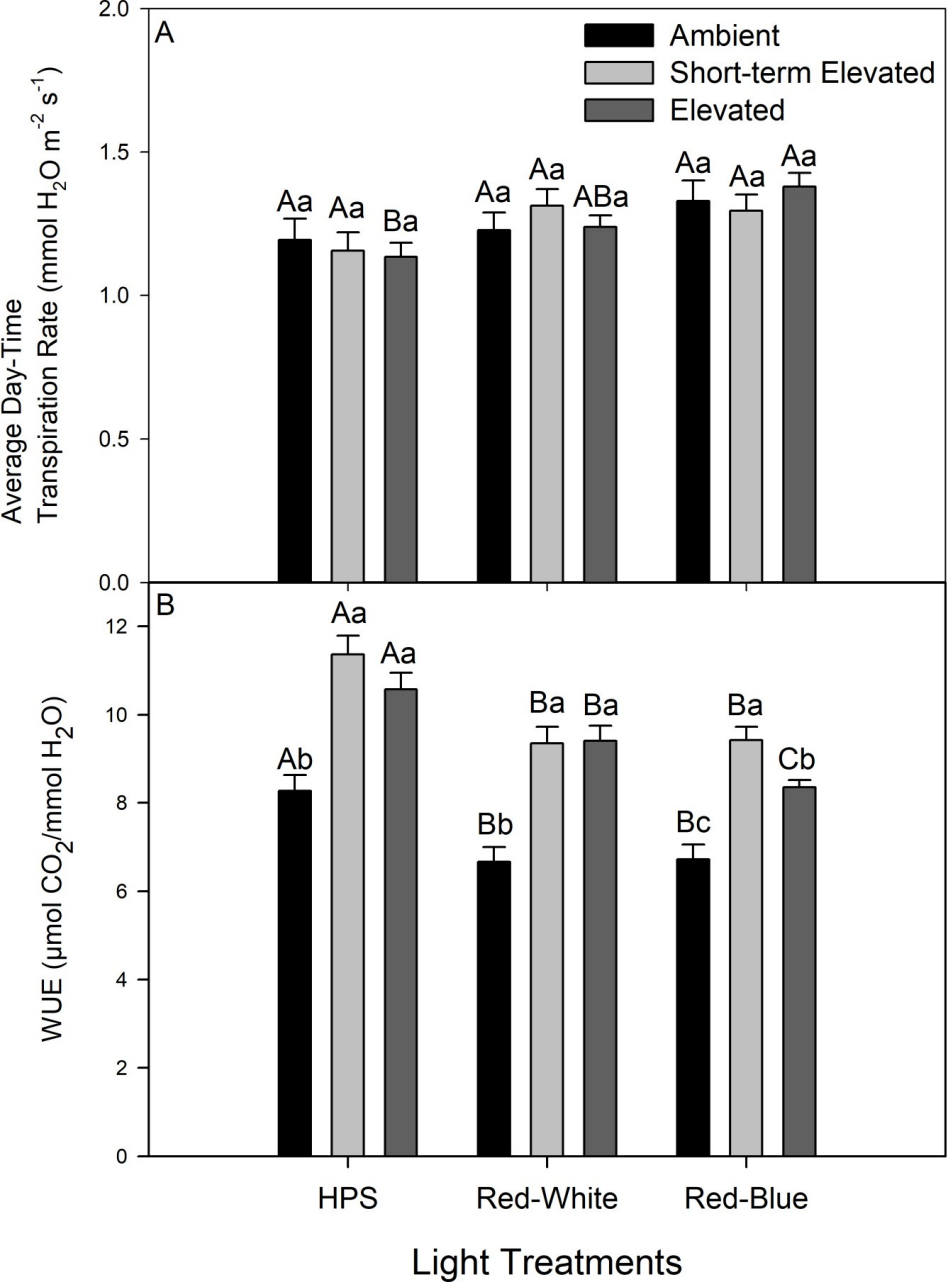
**Average day-time transpiration rates (panel A) and average day-time WUE (panel B).** Different letter groups (A, B, C) represent statistically different values between light treatments at the same CO_2_ analysis conditions within a panel at p<0.05 where n = 14. Different lower-case letter groups represent statistically different values within the same light treatments at different CO_2_ analysis conditions within a panel at p<0.05 where n = 14.

Average day-time transpiration rates were similar under all CO_2_ treatments within a light treatment (Fig 6A). Within the AC treatment and the SEC, all light treatments produced similar average day-time transpiration rates (Fig 6A). Within the EC treatment, plants illuminated with the RB LED produced higher average day-time transpiration rates than plants illuminated with HPS (Fig 6A). Average day-time WUE was greater under illumination with HPS or RW light during EC and SEC conditions compared to AC experiments (Fig 6B). When illuminated with a RB LED, WUE was greater under SEC than other CO_2_ conditions (Fig 6B). Of note, under illumination with a RB LED, plants grown and analyzed under EC produced higher day-time WUE than did plants grown and analyzed under AC (Fig 6B). Importantly, within each CO_2_ condition, plants illuminated with either RB or RW LED produced lower average WUE than did plants under HPS illumination (Fig 6B).

Between the different light treatments, but within CO_2_ conditions, leaf respiration rates were similar (Table 3). When examining within a light treatment, but between CO_2_ treatments, the same is true except for the SEC condition under orange light which produces a lower respiration rate than leaves in the same light treatment under AC conditions (Table 3). Light quality was observed to have no affect on the light compensation point under AC conditions (Table 3). However, under EC treatments, leaves exposed to blue light had a higher light compensation point than all other light treatments except red-blue (Table 3). Similarly, leaves exposed to blue light produced a higher light compensation point under SEC conditions than leaves exposed to either orange or green LEDs (Table 3).

**Table 3:** A summary of the major physiological traits determined by analysis of leaf gas exchange of plant exposed to various CO_2_ and light treatments, as in Figures B and C in S1 File. Respiration values were calculated as the average of 3 replicates when the light level was 0 μmol m^-2^ s^-1^, the light compensation point, and quantum yield were calculated from a regression line (y = mx+b) fitted to the values between the light levels of 0–100 μol m^-2^ s^-1^. The photosynthetic max (Pn_max_) was calculated from f = y_o_+a(1-e^(-b*x)^). Note, quantum yield and Pn_max_ are given on a leaf area basis and a chlorophyll basis to show the difference when expressing data on two different normalization factors.

Light Treatment	CO_2_ Condition	Respiration (μmol CO_2_ m^-2^ s^-1^)	Light Compensation Point (μmol m^-2^ s^-1^)	Quantum Yield (μmol CO_2_ m^-2^ s^-1^/μmol m^-2^ s^-1^)	Pn_max_ (μmol CO_2_ m^-2^ s^-1^)	Quantum Yield (μmol CO_2_ g Chl^-1^ s^-1^/μmol m^-2^ s^-1^)	Pn_max_ (μmol CO_2_ g Chl^-1^ s^-1^)
**HPS**	AC	-1.54(0.33)A	30.61(6.21)A	0.049(0.0006)AB	19.52(3.40)A	0.090(0.001)AB	35.52(6.18)A
	SEC	-1.95(0.24)A	26.49(3.71)AB	0.070(0.0008)A*	24.66(1.98)A	0.13(0.001)A*	44.85(3.60)A
	EC	-1.42(0.22)A	22.56(2.51)B	0.064(0.0006)A*	20.10(0.96)A	0.15(0.001)A*	46.13(2.21)A
**White**	AC	-1.67(0.32)A	20.88(1.95)A	0.056(0.002)A	20.88(1.95)A	0.10(0.004)A	37.98(3.55)A
	SEC	-1.54(0.22)A	24.69(5.13)AB	0.063(0.002)AB*	23.59(0.24)A	0.12(0.003)AB*	42.91(0.44)A
	EC	-1.73(0.09)A	27.81(1.77)B	0.061(0.002)A	23.39(1.98)A	0.14(0.004)A*	53.67(4.55)A*
**Red-Blue**	AC	-2.07(0.20)A	31.28(1.39)A	0.056(0.003)A	20.83(1.19)A	0.096(0.005)A	37.90(2.17)A
	SEC	-1.69(0.06)A	26.59(1.10)AB*	0.059(0.002)AB	23.33(1.96)A	0.11(0.004)AB	42.44(3.56)A
	EC	-1.71(0.21)A	31.59(4.48)AB	0.050(0.002)AB	21.87(0.81)A	0.12(0.005)AB*	50.19(1.86)A*
**Red-White**	AC	-1.82(0.09)A	29.74(1.39)A	0.056(0.003)A	19.79(1.32)A	0.10(0.005)A	36.00(2.41)A
	SEC	-1.78(0.36)A	25.71(4.10)AB	0.063(0.004)AB	23.67(1.49)A	0.12(0.007)AB	43.05(2.71)A
	EC	-1.68(0.08)A	24.29(1.98)B	0.064(0.002)A	20.82(1.01)A	0.15(0.006)A*	47.78(2.32)A*
**Red**	AC	-1.79(0.35)A	33.19(4.69)A	0.052(0.004)A	15.90(0.35)A	0.094(0.007)A	28.97(0.65)A
	SEC	-1.66(0.24)A	31.52(4.42)AB	0.055(0.005)B	23.96(3.07)A*	0.10(0.008)B	43.60(5.58)A*
	EC	-1.64(0.18)A	26.42(4.32)B	0.058(0.004)A	20.63(1.25)A	0.13(0.010)A*	47.34(2.88)A*
**Blue**	AC	-1.29(0.11)A	33.74(3.84)A	0.038(0.002)B	14.45(0.78)A	0.071(0.001)B	26.29(1.39)A
	SEC	-1.89(0.05)A*	36.71(0.87)A	0.052(0.0007)B*	21.31(1.51)A*	0.095(0.001)B	38.76(2.74)A*
	EC	-1.87(0.14)A*	45.93(4.37)A*	0.040(0.001)B	22.28(0.77)A*	0.091(0.003)B*	51.11(1.77)A*
**Orange**	AC	-2.02(0.12)A	37.38(3.54)A	0.052(0.001)A	18.03(1.28)A	0.095(0.003)A	32.80(2.33)A
	SEC	-1.32(0.06)A*	19.20(1.67)B*	0.063(0.002)A*	23.89(1.52)A	0.11(0.003)AB*	43.46(2.77)A
	EC	-1.45(0.22)A*	21.88(3.86)B*	0.057(0.005)A	18.69(2.27)A	0.13(0.011)A*	43.48(5.52)A
**Green**	AC	-1.52(0.14)A	27.67(2.50)A	0.051(0.001)A	18.89(1.09)A	0.093(0.003)A	34.32(1.98)A
	SEC	-1.24(0.16)A	18.40(1.09)B	0.063(0.003)A*	23.05(0.59)A*	0.11(0.006)AB*	41.93(1.07)A*
	EC	-1.54(0.19)A	25.07(4.32)B	0.058(0.003)A	21.62(0.18)A*	0.13(0.006)A*	49.62(0.42)A*

^A, B, C, D^ indicate significant differences (p<0.05) as per multiple means comparison with a Tukey-Kramer adjustment at the same CO_2_ conditions between the different light treatments.

* indicates a significant difference (p<0.05) under the same light treatment between AC (control) CO_2_ condition and either EC or SEC.

Quantum yield is a measure of how much carbon is being fixed based on the amount of light the leaf is exposed to. On a leaf area basis, blue light produced the lowest quantum yield among all light treatments under all CO_2_ conditions (Table 3). Furthermore, under SEC conditions, leaves exposed to red LEDs also produced a lower quantum yield than all other light treatments (Table 3). Different CO_2_ treatments showed no effect on quantum yield in most lights, however, leaves exposed to HPS, white, blue, orange, and green light produced increased quantum yields under the SEC treatment (Table 3). When examining the quantum yield on a chlorophyll basis and considering the intrinsic anatomical differences brought about by acclimation to EC, leaves exposed to EC had higher quantum yields than those under AC under all wavelengths (Table 3). Expressing quantum yield taking on a chlorophyll basis shows that plants under EC conditions are more efficient at fixing carbon than plants under AC conditions.

Under all wavelengths, at each CO_2_ conditions, a similar maximum photosynthetic level was produced on both an area and chlorophyll basis respectively (Table 3). Interestingly, on a chlorophyll basis, in all light treatments except HPS and the orange LED, leaves under the EC condition produced higher maximum photosynthetic levels than leaves under AC conditions (Table 3). These results, considering the change in chlorophyll content due to CO_2_ growth conditions (Table 2), show that there is no photosynthetic acclimation, like that observed when normalizing on leaf area, from leaves grown under EC conditions compared to AC conditions.

Under AC, EC, and SEC CO_2_ conditions, leaves exposed to light from a blue LED produced the lowest photosynthetic rate at a light level of 500 μmol m^-2^ s^-1^ on both a leaf area and chlorophyll basis (Table 4). This result is reflective of the low quantum yield values produced from leaves exposed to blue light, as seen in Table 3. Similar to the results in Table 3, when Pn_500_ was expressed on a chlorophyll basis, leaves under EC conditions produced a higher photosynthetic rate than AC conditions under all light treatments except HPS and green (Table 4). Leaves exposed to white, red-white, red-blue, and green LEDs all showed a decrease in transpiration under SEC compared to AC conditions. Leaves exposed to blue light provided the lowest WUE under AC, SEC, and SAC conditions compared other light treatments (Table 4). This is reflective of leaves exposed to blue light producing both among the highest transpiration rates and the lowest Pn_500_ rates. Consistent with whole plant data, leaves under the SEC condition produced a higher WUE than leaves under AC under all light treatments (Table 4). This result is in part due to the increase in Pn_500_ under SEC compared to AC as well as the reduced transpiration rate brought about by increasing the CO_2_ concentration.

**Table 4:** Leaf CO_2_ and H_2_O gas exchanges under various CO_2_ and light qualities at a light level of 500 μmol m^-2^ s^-1^. Of note, photosynthesis is expressed on both a leaf area and chlorophyll basis.

Light Treatment	CO_2_ Condition	Pn_500_ (μmol CO_2_ m^-2^ s^-1^)	Pn_500_ (μmol CO_2_ g Chl^-1^ s^-1^)	Transpiration_500_ (mmol H_2_O m^-2^ s^-1^)	WUE_500_ (μmol CO_2_ m^-2^ s^-1^/ mmol H_2_O m^-2^ s^-1^)
**HPS**	AC	12.66(1.16)ABC	23.03(2.12)ABC	1.73(0.21)A	7.37(0.43)A
	SEC	17.41(1.69)AB*	31.68(3.08)AB	1.34(0.07)AB	13.14(1.83)B*
	EC	15.35(0.93)AB	35.21(2.13)AB	1.79(0.31)A	8.95(1.20)A
	SAC	12.94(1.62)A	29.69(3.22)A	2.00(0.34)AB	6.60(0.51)BC
**White**	AC	15.68(1.45)A	28.52(2.65)A	2.39(0.20)A	6.57(0.13)A
	SEC	18.07(0.63)AB	32.87(1.16)AB	1.79(0.10)A*	10.29(0.53)B*
	EC	17.45(1.01)A	40.05(2.32)A	1.84(0.13)A	9.60(0.92)A
	SAC	13.29(0.53)A^	30.50(1.21)A^	1.60(0.08)B	8.32(0.27)AB
**Red-Blue**	AC	14.06(0.51)ABC	25.57(0.93)ABC	2.77(0.06)A	5.07(0.18)AB
	SEC	16.55(1.00)AB	30.11(1.83)AB	1.41(0.16)AB*	11.91(0.79)B*
	EC	15.02(0.58)AB	34.47(1.32)AB	2.75(0.40)A	5.68(0.79)A
	SAC	9.79(1.39)A^	22.47(3.19)A^	1.87(0.30)AB^	5.34(0.54)CD
**Red-White**	AC	14.43(0.94)AB	26.25(1.70)AB	2.32(0.08)A	6.21(0.20)A
	SEC	17.14(1.10)AB	31.18(1.99)AB	1.52(0.23)AB*	11.68(1.34)B*
	EC	15.48(0.97)AB	35.52(2.22)AB	1.68(0.24)A	9.71(1.89)A
	SAC	9.48(0.93)A^	22.05(1.76)A^	1.80(0.34)B	5.52(0.51)BC^
**Red**	AC	11.51(0.13)BC	20.95(2.12)BC	1.75(0.16)A	6.69(0.69)A
	SEC	15.64(1.26)AB*	28.45(2.29)AB*	1.54(0.18)B	10.23(0.42)B*
	EC	13.81(1.29)AB	31.69(2.96)AB	1.82(0.10)A	7.57(0.63)A
	SAC	10.48(0.28)A	24.06(0.65)A^	2.45(0.01)AB^	4.21(0.09)CD^
**Blue**	AC	9.98(0.35)C	18.15(0.64)C	2.61(0.50)A	4.06(0.62)B
	SEC	13.51(0.46)B*	24.57(0.84)B*	1.58(0.10)AB	8.64(0.74)B*
	EC	12.74(0.15)B	29.24(0.34)B	2.51(0.36)A	5.30(0.78)A
	SAC	9.79(1.27)A^	22.47(2.92)A^	3.19(0.51)A	3.17(0.44)D
**Orange**	AC	12.70(0.42)ABC	23.10(0.76)ABC	1.82(0.05)A	6.99(0.40)A
	SEC	17.55(0.59)AB*	31.93(1.07)AB*	1.42(0.20)AB	12.89(2.01)B*
	EC	14.38(1.41)AB	33.00(2.62)AB	2.23(0.31)A	6.60(0.51)A
	SAC	11.40(0.46)A^	26.17(1.05)Aa^	2.46(0.24)AB	4.70(0.41)CD
**Green**	AC	14.43(0.83)AB	26.26(1.50)AB	2.36(0.24)A	6.19(0.46)A
	SEC	18.79(1.25)A*	34.18(2.28)A*	0.99(0.06)B*	18.95(0.16)A*
	EC	15.89(0.30)AB	36.46(0.68)AB	1.56(0.26)A	10.32(1.31)A
	SAC	13.94(0.38)A	28.97(3.87)A	1.37(0.07)B	10.23(0.69)A

^A, B, C, D^ indicate significant differences (p<0.05) as per multiple means comparison with a Tukey-Kramer adjustment at the same CO_2_ conditions between the different light treatments.

* indicates a significant difference (p<0.05) between AC (control) and SEC under the same light treatment but between different CO_2_ conditions.

^^^ indicates a significant difference (p<0.05) between EC (control) and SAC under the same light treatment but between different CO_2_ conditions.

**Fig 7:**
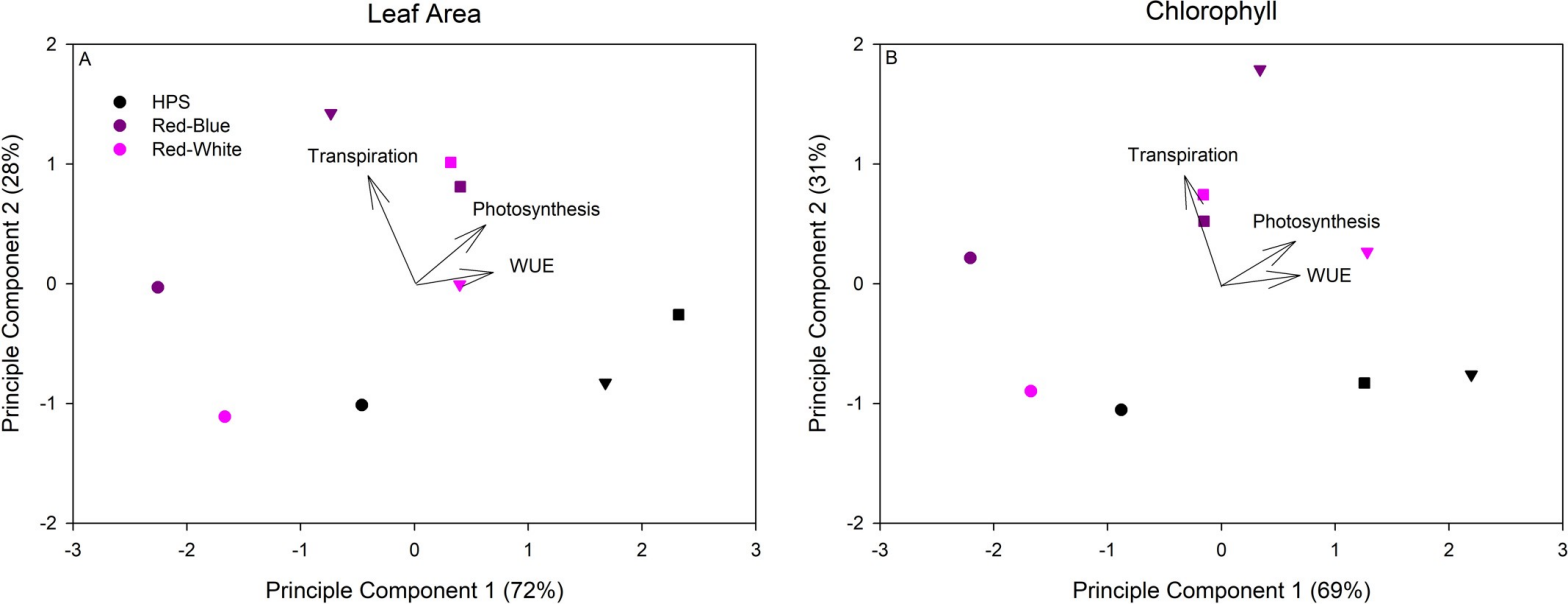
Principle component analysis of the impact of CO_2_ condition and light quality on whole plant gas parameters such as the average photosynthesis, transpiration, and WUE of a tomato at the first flower developmental stage. Values identified by a circle (●) indicates plants grown and analyzed at AC, a triangle (▼) indicates plants grown and analyzed at EC, and a square (■) indicates plants grown at ambient CO_2_ then analyzed under SEC. Panel A represents all values normalized on a leaf area basis. Panel B represents photosynthesis on a chlorophyll basis (μmol CO_2_ g Chl^-1^ s^-1^), transpiration on an area basis (mmol H_2_O m^-2^ s^-1^), and the resulting WUE (μmol CO_2_ g Chl^-1^/ mmol H_2_O m^-2^).

Principle component analysis is a statistical analysis which allows for the assessment of how strongly a set parameter, in this case CO_2_ condition and light quality, affect the response variables (Photosynthesis, transpiration, and WUE). Upon performing a PCA on whole plant data, Fig 7 was obtained. PCA was performed on each individual run (Figure D in S1 File), however, for simplicity, Fig 7 only shows the average values. Values associated with EC and SEC (i.e., Triangles and squares) experiments tend to associate more with the photosynthesis and WUE vectors, independent of light quality (Fig 7A). Of note, values associated with plants illuminated with RB and RW LEDs under all CO_2_ conditions are more closely associated with the transpiration vector (Fig 7A). Taken together, these results indicate that transpiration rate is controlled more so by spectral quality while photosynthesis and WUE are more influenced by elevations in the CO_2_ concentration (Fig 7A). When expressing photosynthetic data on a chlorophyll basis, EC conditions produce a stronger influence on photosynthesis and WUE (Fig 7B) than when photosynthesis is expressed on a leaf area basis (Fig 7A). For example, note the rightward shift of the triangular symbols in Fig 7B. The shift to the right due to EC exposure indicates the importance of considering changes in plant anatomy and biochemistry when analyzing primary gas exchanges.

**Fig 8:**
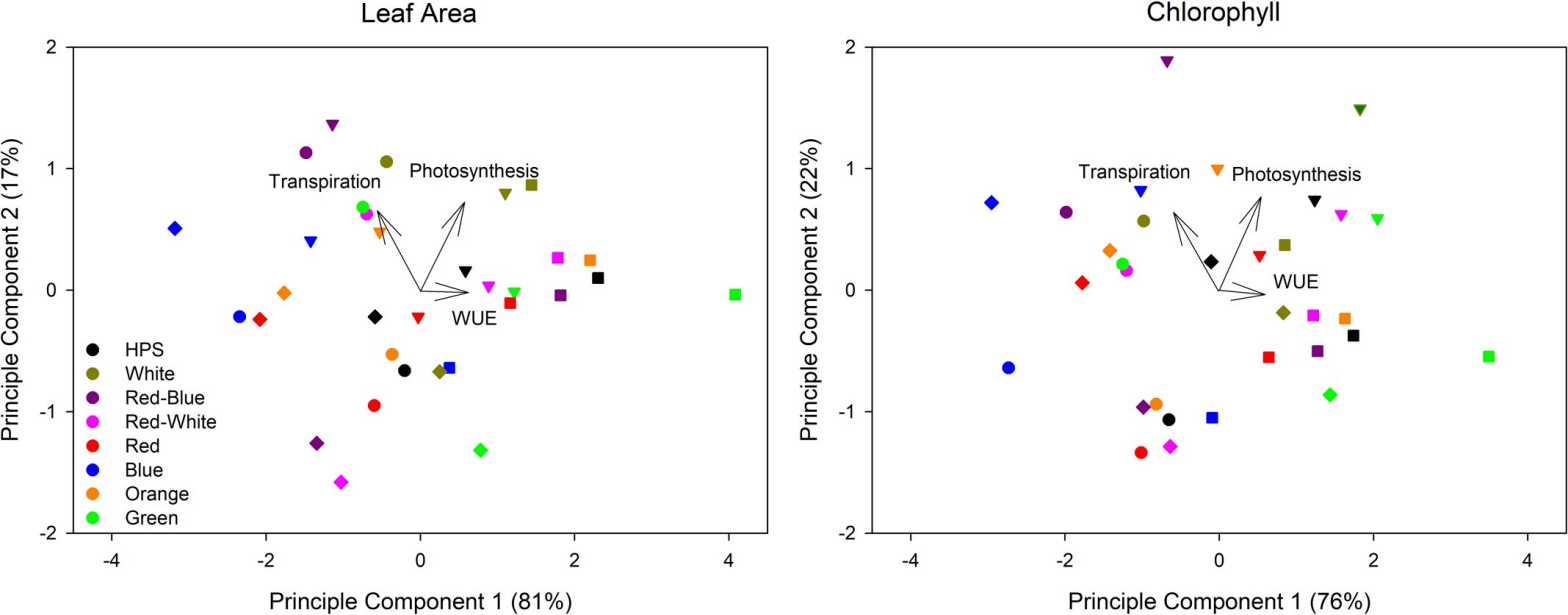
Principle component analysis of the impact of CO_2_ condition and light quality on leaf gas parameters such as average photosynthesis, transpiration, and WUE of a tomato at the first flower developmental stage and a light level of 500 μmol m^-2^ s^-1^. Values identified by a circle (●) indicates plants grown and analyzed at AC, a triangle (▼) indicates plants grown and analyzed at EC, a square (■) indicates plants grown at ambient CO_2_ then analyzed under SEC, a diamond indicates plants grown under elevated CO_2_ then analyzed under SAC. Panel A represents all values normalized on a leaf area basis. Panel B represents photosynthesis on a chlorophyll basis (μmol CO_2_ g Chl^-1^ s^-1^), transpiration on an area basis (mmol H_2_O m^-2^ s^-1^), and the resulting WUE (μmol CO_2_ g Chl^-1^/ mmol H_2_O m^-2^).

Similar to whole plant data, a PCA was performed on all experimental runs of leaf data at a light level of 500 μmol m^-2^ s^-1^ (Figure E in S1 File), and for ease of interpretation, the averages are displayed in Fig 8. Results from the PCA involving leaf data are consistent with that in Fig 7 (Fig 8). On a leaf area basis, plants exposed to EC or SEC conditions tend to affect both photosynthesis and WUE more than transpiration (Fig 8A). Furthermore, consistent with Fig 7B, when leaf photosynthesis was expressed on a chlorophyll basis, plants under the EC conditions tend to affect the photosynthetic rate and WUE more than when expressed on a leaf area basis (Fig 8B). Again, the data presented in Fig 8B emphasizes the importance of recognizing key anatomical and biochemical differences brought about by acclimation to different CO_2_ treatments.

## Discussion

The effect of growing plants under EC on flowering time is highly variable among species [2, 31]. For tomato, growth under EC generally results in a decrease in the time to flowering [32–33] which is confirmed by the 8-day decrease observed in our study. Furthermore, growth under EC increases biomass gain within tomatoes [6]. In this study, we have purposely compared plants at a similar developmental stage when grown under the different CO_2_ levels. Doing so, an increase in total dry biomass was not apparent, likely due to the age difference (Fig 1). However, at the first flower developmental stage, an increase in root biomass was observed when plants were grown under EC which is consistent with results from many species including tomato (Fig 1) [34–36]. An increase in root biomass could be associated with an increase in plant biomass gain at similar ages, generally observed due to the ability of a plant to uptake more water and nutrients needed for growth [37].

Consistent with the effects of long-term growth under EC, tomato plants exhibited an increase in stem elongation compared to AC grown plants (Table 1) [20]. Plants grown under EC produced less leaf area than those grown under AC conditions which opposes the results from Ho (Table 1) [38]. However, plants in our study were analyzed at the same developmental stage and thus intrinsically different ages which could account for the differences in results observed. Furthermore, an increase in specific leaf weight was observed by plants grown under EC (Table 1). An increase is specific leaf weight is consistent with literature and due to an increase of non-structural carbohydrates, particularly starch, at EC levels which accumulate within the leaves [39].

Chlorophyll *a*, chlorophyll *b*, and total chlorophyll, elements involved in light absorption, all decreased in plants grown under EC (Table 2). Similar results were observed in tomato and *Trifolium subterraneum* [40–41]. It has been proposed that an increase in CO_2_ levels leads to a degradation of chloroplast as a result of the excess starch accumulation [40]. However, other literature in various species has shown no significant effect of CO_2_ concentration on chlorophyll content [42–43]. Thus, while results presented in Table 2 clearly indicate decreases in chlorophyll and carotenoid content, the literature surrounding this topic is still variable.

Whole plant NCER under all CO_2_ conditions show no difference due to spectral quality which confirms results published in Lanoue et al. (Fig 2) [15]. This result indicated that when considering the complexity of a whole plant canopy, short-term exposure to wavelength specific lighting is unable to affect the primary photosynthetic and respiratory processes. Interestingly, day-time NCER decreases in the latter part of the day under all CO_2_ conditions but is observed to decrease more when plants were exposed to SEC (Fig 2). A decrease in photosynthetic rate has also been observed in rice and was observed to be more dramatic with an increased CO_2_ concentration [44]. It has been suggested that under high carbohydrate production, such as the conditions used in EC and SEC experiments, an end product feedback causes an inhibition of the photosynthetic apparatus resulting in the drop off in NCER later in the photoperiod (Fig 2) [45–47].

When plants were grown under AC than exposed to SEC an increase in average whole plant photosynthesis was observed compared to plants grown and analyzed under AC (Fig 4). Short-term exposure to elevated CO_2_ leading to an increase in the photosynthetic rate is attributed to the increase of the carboxylation reaction of RuBisCO and subsequent decrease in photorespiration [48–50]. Under long-term acclimation to EC, after an initial increase in photosynthesis, a decrease is reported in tomato when compared to plants grown under AC indicating acclimation of the photosynthetic machinery to EC [22]. However, it is important to note that plants grown under different CO_2_ conditions have different anatomy and biochemical features, specifically, different chlorophyll content on a leaf area basis (Table 2). Therefore, when normalizing photosynthetic rates on a chlorophyll basis, plants grown under EC produced approximately a 52% increase in the photosynthetic rate under all light treatments compared to the ambient grown plants (Fig 4G).

Night-time respiration rates are associated with day-time photosynthetic rates and carbohydrate status [51–52]. This association is clearly indicated by results in Fig 4B where plants analyzed under SEC produced higher average photosynthetic rates and subsequent higher respiration rates than did plants with a lower photosynthetic rate. However, when normalizing on both leaf area and dry biomass, this relationship is not observed (Fig 4D and 4F). Thus, the effect of CO_2_ concentrations on night-time respiration is still debateable as both increases and reductions have been reported elsewhere [53]. Importantly, whole plant night-time respiration rates are not observed to be affected by day-time light quality which confirm previous results for tomato reported by Lanoue et al. [15].

While both CO_2_ and H_2_O gas exchange with the external environment are mediated via stomata, transpiration control is also linked to cryptochrome (CRY), a blue light receptor, which is known to follow an entrained circadian rhythm [54]. Furthermore, the diurnal patterns of CRY follow closely to that of transpiration, indicating a strong link between CRY activation and transpiration rates [55–56]. Thus, the results in Fig 5 indicate that all light treatments provide adequate amounts of blue light to maintain normal circadian rhythms of stomatal behaviour.

Whole plant WUE describes the relationship between water loss via transpiration and carbon fixation via photosynthesis of a canopy (Fig 6B). Plants grown under AC were exposed to SEC, a decrease in WUE was observed in the latter part of the photoperiod (Fig 5E). This decrease is due to the drastic reduction in photosynthetic rate under this condition, something which is not as strongly apparent in AC or EC conditions, coupled with the entrained circadian rhythm of transpiration rate (Figs 2 and 5). As mentioned above, the reduction in the photosynthetic rate is consistent with feedback inhibition via plant carbon status and thus able to affect WUE more so during SEC challenge than AC or EC conditions [45–47].

Decreases in WUE were observed when plants were illuminated with either RB or RW LEDs compare to illumination with HPS under all CO_2_ condition (Fig 6B), confirming results first noted at AC [15]. Water-use-efficiency results indicate that plants grown under EC and those grown under AC but analyzed under SEC fix more carbon per water loss via transpiration than plants under AC (Fig 6B). Due to the similarity of day-time transpiration rates among CO_2_ conditions, it follows that the increase in WUE under EC and SEC are predominantly due to an increase in photosynthetic rates.

Average day-time transpiration rate under AC were unaffected by spectral quality, as were rates of plants analyzed under SEC (Fig 6A). Plants grown and analyzed under EC which were illuminated with a RB LED produced higher average day-time transpiration rates than plants illuminated with HPS lighting (Fig 6A). This result is attributed to the increased amount of blue light emitted by the RB LED which is known to cause stomatal opening [57–59]. It is noteworthy that the whole plant chambers used during our experiments control air temperature more tightly than in a greenhouse. Thus, it is unlikely that excess heat generated by HPS lighting will affect our gas exchange measurements as it may in greenhouses equipped with HPS lamps.

It is well known that photosynthesis and transpiration are tightly linked as they both rely on stomatal opening [60]. In general, under all wavelengths of light tested, leaf transpiration rates were reduced by short-term exposure to elevated CO_2_, consistent with literature [61]. However, contrary to leaf results from FACE experimentation indicating a decrease in transpiration rates under EC [61–63], no differences were observed during whole plant H_2_O gas exchange analysis under increased CO_2_ (Fig 6A). However, similar transpiration rates from plants exposed to different CO_2_ conditions has been previously observed. Using lisianthus plants grown under AC conditions, whole plant transpiration rates were similar when analyzed under AC and SEC conditions (data unpublished). Similarly, wheat leaves which were exposed to elevated CO_2_ produced similar stomatal conductance values to leaves exposed to ambient CO_2_ over a wide range of light intensities [64]. In *Arabidopsis thaliana* plants grown under both AC and EC conditions, whole plant transpiration rates were also observed to be similar [17]. It is noteworthy to bring to the readers attention that when expressed on a plant basis, plants acclimated to EC would have a lower transpiration rate compared to plant grown under AC. However, due to differences brought about during growth under different CO_2_ conditions (Table 1), when expression transpiration data on the traditional leaf area basis, this difference is negated due to the difference in leaf area.

The discrepancy between the response of leaf and whole plant transpiration resulting from exposure to SEC is likely due to the additional complexity involved in measurements of whole plant gas exchange. Measuring whole plant transpiration (Fig 5) shows the complicated nature of simple extrapolation of leaf data (Table 2) as it does not account for differences in leaf age, canopy architecture, micro-climate, and mutual shading [65]. During leaf level measurements, controlling leaf temperature can be done accurately. however, controlling canopy temperature can be more difficult during whole plant measurements. During SEC, it is possible that the leaf temperature of the plants was increased compared to plants under AC [66]. The reaction will then be to increase transpiration in order to regulate the leaf temperature. It can be hypothesized that under SEC, the increase in leaf temperature is able to compensate for the affect of high CO_2_ on stomatal function, leading to a higher whole plant transpiration rate than one would expect when modelling using solely leaf data. Furthermore, mature leaves under low light intensity, such as those deep within the canopy, naturally transpire at a lower rate. Thus, differences in transpiration rate between AC and SEC conditions will be minimal. The complexity brought about by measuring a whole plant instead of merely a leaf, illustrates the difference between CO_2_ and H_2_O gas exchange via stomata of newly formed and mature leaves under different environmental parameters.

A principle component analysis was performed to assess the effects of changes in the light spectrum and/or CO_2_ conditions on photosynthesis, transpiration, and WUE (Fig 7). The analysis determines that values associated with EC plants and those analyzed under SEC are more closely associated with changes in photosynthesis and WUE (Fig 7). In contrast, these same points are observed to have a relatively small influence on transpiration rates (Fig 7). Fig 7 indicates that both RB and RW LEDs affect transpiration rates more than illuminating plants with HPS lighting. These results on whole plant CO_2_ and H_2_O gas exchange confirm leaf studies of *Xanthium strumarium* L. which showed a lower stomatal sensitivity to increasing CO_2_ concentrations [67]. Furthermore, PCA performed on leaf data shows similar results with plant exposed to elevated CO_2_ levels tending to affect photosynthesis and WUE more than transpiration (Fig 8). The affects of both light quality and CO_2_ concentration have on transpiration and WUE have implication in water and nutrient management during winter greenhouse production when humidity is generally low, inhibiting stomatal function [68–69].

Taken together, results presented above show evidence that plants acclimated to EC and those exposed to SEC produce higher photosynthetic rates while having similar whole plant transpiration values compared with plants under AC during all light treatments. Furthermore, illumination with either RB or RW LED produced lower WUE than illumination with HPS luminaries, likely due to a higher blue light component. Results presented here allow for a greater understanding of the inter-play between a common greenhouse production method, CO_2_ enrichment, and a new, fast-evolving technology, wavelength specific LED lighting. By doing such research on a whole plant, a better understanding of the physiological response can be obtained while considering leaf age and canopy architecture, factors which are not fully taken into account when conducting leaf level measurements. This information can lead to the optimization of lighting strategies as well as watering and fertilizing regimes in greenhouse production providing increased sustainability and yield.

## Supporting information

S1 File **Figure A. Photosynthetically active radiation spectrum of HPS, RB LED, and RW LED lights used during the whole plant NCER experiment (Panel A)**. Photosynthetically active radiation spectrum of white, red-blue, red-white, red, blue, orange, and green LEDs as well as HPS lighting used during the leaf NCER experiment. Each light spectrum was determined using a spectroradiometer (Flame Spectrometer, Ocean Optics, Dunedin, FL, USA). **Figure B. Leaf NCER of tomato plants under AC (A), SEC (B), and EC (C) exposed to various spectral qualities expressed on an area basis**. The regression lines are fit to f = y_o_+a(1-e^(-b*x)^) where y_o_ is the respiration rate at a light level of 0 μmol m^-2^ s^-1^, a is the maximum photosynthetic rate (μmol CO_2_ m^-2^ s^-1^), and b is a constant. Each regression line is fitted to n = 3 leaves. **Figure C. Leaf NCER of tomato plants under AC (A), SEC (B), and EC (C) exposed to various spectral qualities expressed on a chlorophyll basis**. The regression lines are fit to f = y_o_+a(1-e(^-b*x)^) where y_o_ is the respiration rate at a light level of 0 μmol m^-2^ s^-1^, a is the maximum photosynthetic rate (μmol g Chl^-1^ s^-1^), and b is a constant. Each regression line is fitted to n = 3 leaves. **Figure D. Principle component analysis of the impact of CO_2_ condition and light quality on whole plant gas parameters such as photosynthesis, transpiration, and WUE of a tomato at the first flower developmental stage**. Values identified by a circle (•) indicates plants grown and analyzed at AC, a triangle (▲) indicates plants grown and analyzed at EC, and a square (■) indicates plants grown at ambient CO_2_ then analyzed under SEC. Panel A represents all values normalized on a leaf area basis. Panel B represents photosynthesis on a chlorophyll basis (μmol CO_2_ g Chl^-1^ s^-1^), transpiration on an area basis (mmol H_2_O m^-2^ s^-1^), and the resulting WUE (μmol CO_2_ g Chl^-1^/ mmol H_2_O m^-2^). **Figure E. Principle component analysis of the impact of CO_2_ condition and light quality on leaf gas parameters such as photosynthesis, transpiration, and WUE of a tomato at the first flower developmental stage and a light level of 500 μmol m^-2^ s^-1^**. Values identified by a circle (•) indicates plants grown and analyzed at AC, a triangle (▼) indicates plants grown and analyzed at EC, a square (■) indicates plants grown at ambient CO_2_ then analyzed under SEC, a diamond indicates plants grown under elevated CO_2_ then analyzed under SAC. Panel A represents all values normalized on a leaf area basis. Panel B represents photosynthesis on a chlorophyll basis (μmol CO_2_ g Chl^-1^ ^s-1^), transpiration on an area basis (mmol H_2_O m^-2^ s^-1^), and the resulting WUE (μmol CO_2_ g Chl^-1^/ mmol H_2_O m^-2^)
(DOCX)
