# Supplementary figures and images for: Effect of elevated CO2 and spectral quality on whole plant gas exchange patterns in tomatoes
Source: PLoS One. 2018 Oct 18;13(10):e0205861. doi: 10.1371/journal.pone.0205861 (PMC6193678; doi:10.1371/journal.pone.0205861)

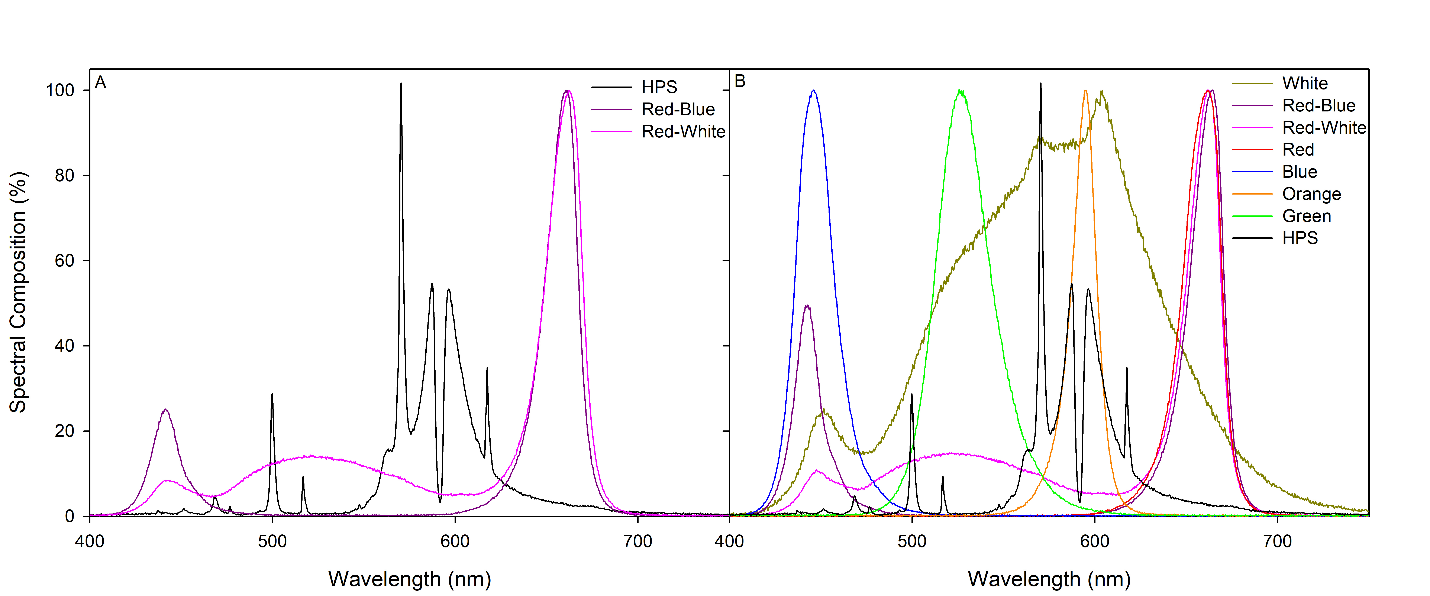


**Figure A**


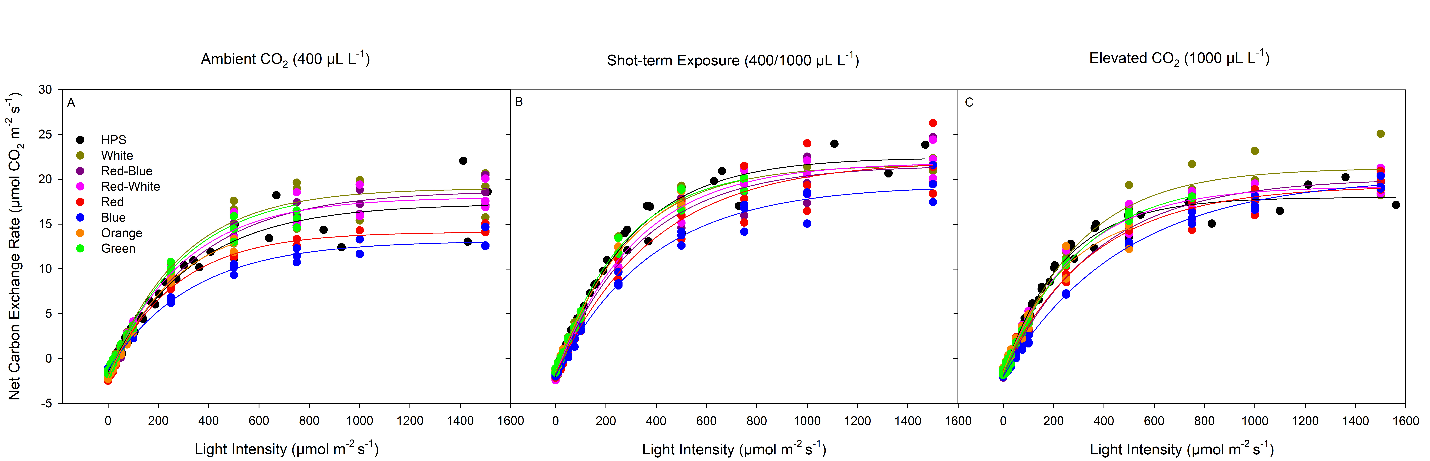


**Figure B**


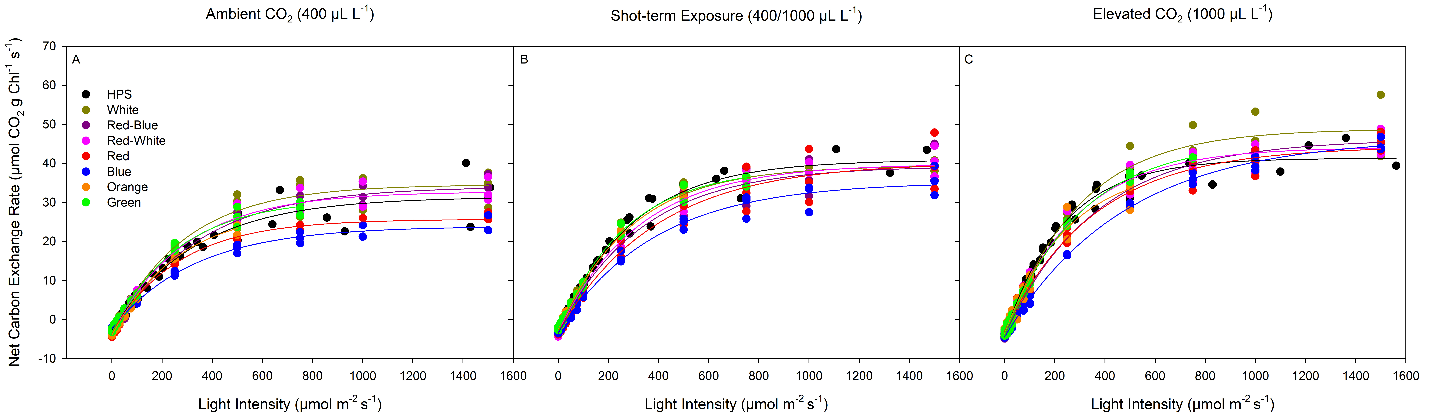


**Figure C**


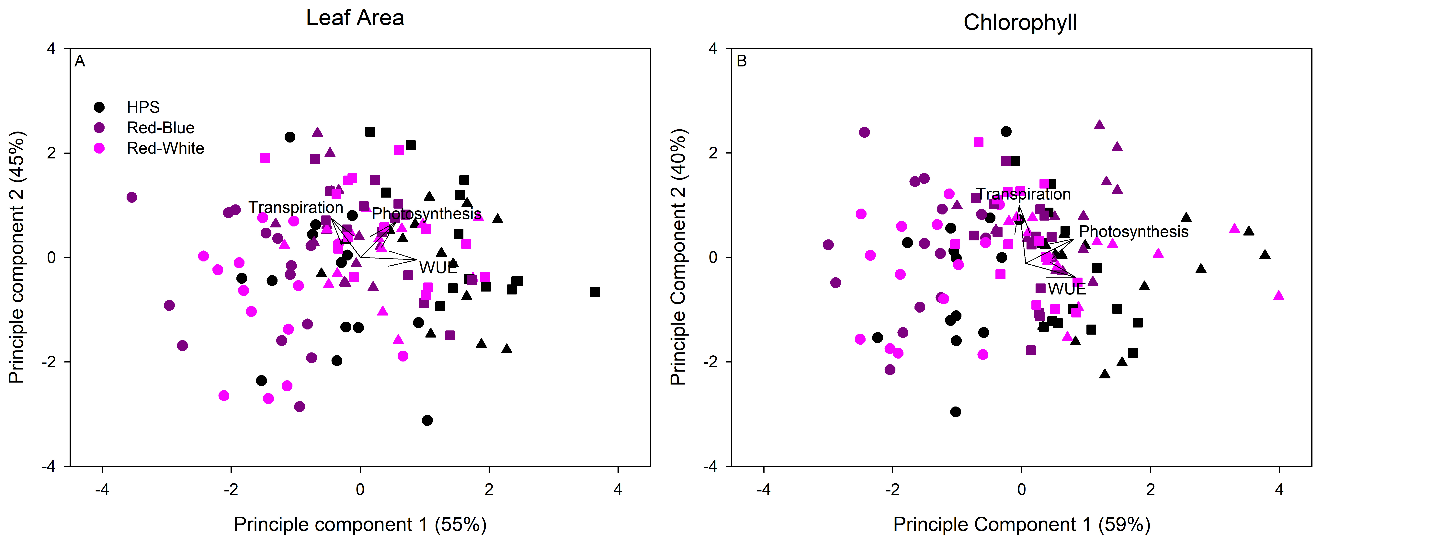


**Figure D**


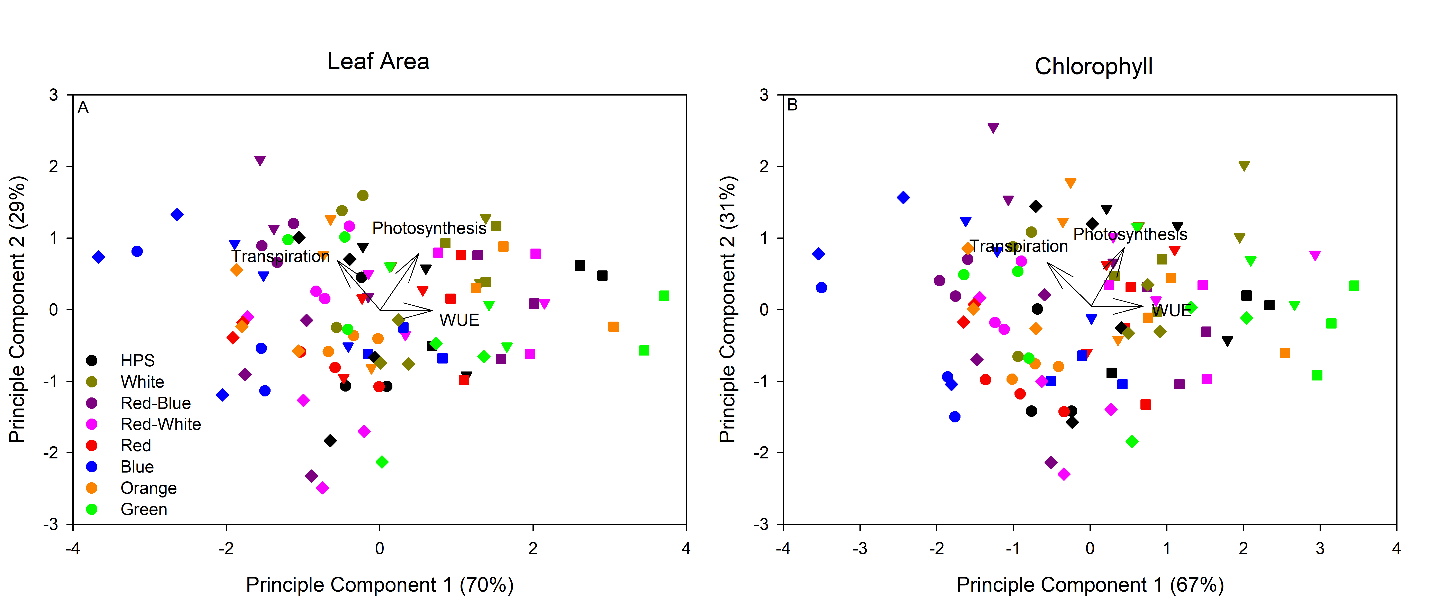


**Figure E**

Supplement: S1 File — Figure A. Photosynthetically active radiation spectrum of HPS, RB LED, and RW LED lights used during the whole plant NCER experiment (Panel A). Photosynthetically active radiation spectrum of white, red-blue, red-white, red, blue, orange, and green LEDs as well as HPS lighting used during the leaf NCER experiment. Each light spectrum was determined using a spectroradiometer (Flame Spectrometer, Ocean Optics, Dunedin, FL, USA). Figure B. Leaf NCER of tomato plants under AC (A), SEC (B), and EC (C) exposed to various spectral qualities expressed on an area basis. The regression lines are fit to f = yo+a(1-e(-b*x)) where yo is the respiration rate at a light level of 0 μmol m-2 s-1, a is the maximum photosynthetic rate (μmol CO2 m-2 s-1), and b is a constant. Each regression line is fitted to n = 3 leaves. Figure C. Leaf NCER of tomato plants under AC (A), SEC (B), and EC (C) exposed to various spectral qualities expressed on a chlorophyll basis. The regression lines are fit to f = yo+a(1-e(-b*x)) where yo is the respiration rate at a light level of 0 μmol m-2 s-1, a is the maximum photosynthetic rate (μmol g Chl-1 s-1), and b is a constant. Each regression line is fitted to n = 3 leaves. Figure D. Principle component analysis of the impact of CO2 condition and light quality on whole plant gas parameters such as photosynthesis, transpiration, and WUE of a tomato at the first flower developmental stage. Values identified by a circle (•) indicates plants grown and analyzed at AC, a triangle (▲) indicates plants grown and analyzed at EC, and a square (■) indicates plants grown at ambient CO2 then analyzed under SEC. Panel A represents all values normalized on a leaf area basis. Panel B represents photosynthesis on a chlorophyll basis (μmol CO2 g Chl-1 s-1), transpiration on an area basis (mmol H2O m-2 s-1), and the resulting WUE (μmol CO2 g Chl-1/ mmol H2O m-2). Figure E. Principle component analysis of the impact of CO2 condition and light quality on leaf gas parameters [file pone.0205861.s001.docx]
